# Mapping needles, reducing harm: Findings from a geospatial, community-based needle collection and naloxone training initiative in Saskatchewan, Canada

**DOI:** 10.1186/s12954-026-01477-z

**Published:** 2026-06-04

**Authors:** Nelson Pang, Shiny Mary Varghese, Vidya Dhar Reddy, Tashia Acoose, Erin Hidlebaugh, Sandra Kwan, Megan Rowe, Priscilla Medeiros, Paul A. Shuper, Francisco Ibáñez-Carrasco, Daniel Grace, Andrew D. Eaton

**Affiliations:** 1 Eaton Lab, Faculty of Social Work, University of Regina, Regina, SK Canada; 2 Factor-Inwentash Faculty of Social Work, University of Toronto, Toronto, ON Canada; 3 AIDS Programs South Saskatchewan, Regina, SK Canada; 4 Edwin S.H. Leong Centre for Healthy Children, University of Toronto, Toronto, Canada; 5 Centre for Addiction and Mental Health, Toronto, ON Canada; 6 Department of Psychiatry, University of Toronto, Toronto, ON Canada; 7 Dalla Lana School of Public Health, University of Toronto, Toronto, ON Canada; 8 Jane Addams College of Social Work, University of Illinois Chicago, 1040 W Harrison St, IL Chicago, USA

**Keywords:** Harm reduction, Naloxone training, Geospatial, injection drug use

## Abstract

**Background:**

The opioid crisis is a major public health issue in Canada, with prairie provinces such as Saskatchewan experiencing particularly high rates of opioid-related harms. Factors contributing to this crisis include an unstable drug supply, limited access to harm reduction services, and structural challenges such as poverty and housing instability. The rise of fentanyl has further exacerbated overdose risks, particularly among people who use drugs. Harm reduction programs, such as opioid overdose education and naloxone distribution, have proven effective in reducing overdose fatalities and improving community health. While geospatial analysis has shown promise in identifying areas of high need for targeted harm reduction interventions, its integration into harm reduction strategies remains underexplored.

**Methods:**

This study utilized data from 44 participants who completed pop-up naloxone training sessions in Regina, Saskatchewan, between August 2023 and September 2024. Data sources include geospatial information on discarded needles from the ReportNeedles.ca platform and survey responses evaluating opioid overdose and naloxone administration using a modified Opioid Overdose Knowledge Scale. Naloxone training sessions were targeted to areas with a high number of discarded needles determined by the ReportNeedles.ca platform. Geospatial analyses were conducted using ArcGIS to map needle prevalence and assess the accessibility of harm reduction services based on walk-time buffers.

**Results:**

Between August 2023 and August 2024, 315 reports on ReportNeedles.ca led to the disposal of 2,836 needles. Geospatial analysis revealed clustering of discarded needles in Regina’s city center, with some seasonal variation. Pop-up training sites expanded the accessibility of naloxone services, with 70% of participants reporting living within a 15-minute walk to pop-up Naloxone trainings. However, geospatial analysis revealed gaps in service accessibility specifically in suburban areas. Participants in pop-up naloxone trainings demonstrated strong knowledge of overdose recognition and naloxone administration.

**Conclusions:**

This study demonstrates potential benefit in integrating geospatial analysis with harm reduction interventions to address the opioid crisis. By identifying needle prevalence hotspots and utilizing pop-up naloxone training, service providers can improve service accessibility.

## Background

The opioid crisis is a major public health issue globally, especially in Canada, the United States and Europe [1–4]. Between 2019 and 2021, the annual number of opioid-toxicity deaths in Canada more than doubled, comprising more than 25% of deaths among young Canadians [5]. The opioid overdose crisis in Canada has resulted in a substantial increase in deaths, hospital admissions, and blood-borne infections associated with substance use [6, 7]. While fatal overdoses have sharply risen nationwide, Western Canada has been predominantly impacted by this epidemic [5]. In particular, Saskatchewan has reported a nearly 300% increase in opioid-toxicity deaths between 2010 and 2020 [8]. The rise in overdose rates in Saskatchewan has been further accelerated by reduced access to harm reduction services and an increasingly unstable, illicit drug market [5]. The high rates of opioid-related harms, including illness, hospitalization, and death, underscore the urgent need for expanded access to healthcare, social services, and harm reduction programs.

The risks associated with opioid use are particularly acute for people who use drugs (PWUD), especially people who inject drugs. Research suggests that approximately one-in-five people who inject drugs is at risk of a non-fatal overdose each year [9]. The factors that mediate risk of overdose are complex and multifaceted, encompassing social, biological, political, and environmental dimensions. Structural vulnerabilities, such as poverty, housing instability, punitive policies (e.g., criminalization of drug use, restriction on harm reduction programs), and historical oppression, limit a person’s ability to engage in risk-reduction practices (e.g., needle exchange) and access interventions (e.g., substance use treatment) [10]. Additionally, people who experience incarceration, homelessness, and sex work are disproportionately affected by poorer physical and psychological health outcomes [11]. In many cases, substance use becomes a coping mechanism for these challenging conditions, furthering the increased risk of overdose [12].

Indigenous Peoples in Canada are disproportionately affected by opioid related harms, including overdose mortality and HIV/STBBI transmission, due to intersecting structural inequities rooted in colonialism, systemic racism, intergenerational trauma, and ongoing barriers to healthcare access [8, 13, 14]. In Saskatchewan, these inequities are particularly pronounced, with Indigenous communities experiencing higher rates of drug toxicity deaths and related health outcomes compared to non-Indigenous populations [8]. Regina is a mid-sized prairie city with spatially concentrated social and health inequities, including disproportionate impacts of poverty, housing instability, and drug-related harms [15]. As of September 2025, there are seven urban Indigenous reserves in Regina [16]. Urban reserves in Saskatchewan influence patterns of service access, community engagement, and the demographic composition of populations reached through harm reduction interventions [17].

The COVID-19 pandemic exacerbated the vulnerabilities faced by people who use drugs. Specifically, the public health measures during the COVID-19 pandemic may have led to a more adulterated, illicit drug supply [5, 18]. Border closures, which disrupted the supply of conventional methamphetamines and opioids, have reportedly resulted in greater contamination of illicit drugs with synthetic opioids, specifically fentanyl [18]. Fentanyl is rapid-acting yet short-lasting, which in combination with high potency and an extremely narrow margin of safety, increases risk of addiction, withdrawal, and overdose [19]. While many people who use drugs are aware of fentanyl and its risks, this population has little agency in detecting and avoiding fentanyl-adulterated substances due to an unpredictable drug supply [20, 21]. For example, in a study of 313 people who inject drugs in New York City, only 20% of participants reported recent, intentional fentanyl use, but 83% of participants tested positive for fentanyl [20]. Repeated, unintentional exposure to fentanyl may lead to physical dependence on the opioid and aversion to drugs with higher margins of safety, resulting in increased risk of overdose and other opioid-related harms [20].

In response to the rise of opioid-related overdoses and deaths, harm reduction strategies have become essential public health interventions aimed at reducing the negative impacts of opioid use. As part of overdose prevention efforts, opioid overdose prevention education and naloxone distribution (OEND) programs have been developed. These brief interventions commonly detail the risk factors for overdose, signs and symptoms of an opioid overdose, steps to take in an overdose, and instructions on how to appropriately administer naloxone, which is subsequently accompanied by the provision of a naloxone kit to the participant [22]. Naloxone is an opioid antagonist that reverses opioid-induced respiratory depression and prevents fatal overdose by displacing opioid agonists from receptor sites [23, 24]. The literature has demonstrated that naloxone training initiatives produce successful outcomes at the individual and community levels [23–27].

Numerous studies have highlighted the efficacy of OEND programs in providing people who use drugs and the general public knowledge to respond effectively to overdose [23, 25–27]. For example, Winhusen et al., reported that a harm reduction intervention increased knowledge of overdose, decreased perceived treatment barriers, and reduced overdose-risk behaviours [28]. Similarly, Gaston et al., found that naloxone training significantly boosted participants’ confidence and willingness to intervene in overdose situations, with many reporting knowledge retention and a willingness to train others in overdose management months after the intervention [22]. PWUD have additionally reported improvements in personal health, safety, and self-efficacy after receiving OEND training [29]. Likewise, prior research suggests that greater feelings of hope elicited by the ability to reverse overdose has also been linked to willingness to engage in Human Immunodeficiency Virus (HIV) and Hepatitis C testing, demonstrating the substantial beneficial effects of naloxone training on high-risk individuals [29]. Beyond individual benefits, population-level impacts of naloxone programs have been observed. Communities implementing OEND initiatives have experienced substantial reductions in fatal overdose rates [29, 30]. Maxwell et al., reported that the implementation of naloxone training initiatives in Cook County, Chicago reversed a trend of increasing heroin overdose deaths and reduced overdose fatalities by 20% [29]. Overall, naloxone training programs pose considerable benefits for individuals and communities, particularly people who use drugs.

## Geospatial analysis and harm reduction

Geographic information systems (GIS) can capture, store, analyze, and display spatial data [26]. Geospatial analysis is a vital tool for visualizing health research, due to its ability to examine associations between location, environment, and disease [31]. By mapping disease clusters in a given community, GIS can inform targeted investigations, allowing resources and interventions to be strategically deployed where they are most needed [31].

In the context of the opioid crisis in North America, geospatial analysis has been employed to study spatial and demographic trends of opioid-related overdose. For example, Des Jarlais et al., identified hotspots for drug overdose and HIV transmission in New York City by mapping participants’ geographic patterns of high-risk behaviour according to zip or postal codes [32]. On a larger scale, several American studies have utilized zip code data in electronic medical record databases to decipher areas with elevated opioid-related healthcare visits, opioid poisoning diagnoses, and emergency medical service use, improving understanding of geographic healthcare utilization patterns [33–35]. Outside of tracking opioid-toxicity deaths, geospatial analysis can be equipped to analyze the distribution of harm reduction resources in a given area. Wong et al. measured the distance between the residences of methadone clinic clients and treatment centres in Hong Kong to assess the spatial distribution of patients and their access to treatment [36]. Similarly, Abell-Hart et al. calculated the distance between opioid-overdose patients’ addresses to the nearest pharmacy that provided naloxone and buprenorphine [37]. The authors discovered that areas without nearby naloxone-dispensing pharmacies significantly overlapped with the locations of many opioid-overdose patients. However, geospatial analysis based on patients’ zip codes may be insufficient in determining target sites for prevention efforts [37]. People do not necessarily use opioids where they live, therefore targeting resources to home addresses may fail to amend a community’s opioid-related risk [33]. Furthermore, many people who use drugs are experiencing homeless or transient, resulting in the undercounting and exclusion of this population under conventional address-based analysis [35, 37]. Consequently, innovative methods are needed to accurately deliver resources to marginalized, high risk populations.

### Project ReportNeedles.ca

ReportNeedles.ca is a web-based platform developed by AIDS Programs South Saskatchewan (APSS) in Canada that was designed to address challenges surrounding community needle prevalence. APSS operates in Regina, Saskatchewan and provides services for HIV/AIDS, Hepatitis C, and harm reduction through education, prevention, support and care. While other health and social services exist in Regina, APSS is a low barrier program that is accessed by socially marginalized groups including people who use drugs. Launched in April 2021, the platform allows community members to report the presence of discarded needles in public spaces. In fall 2022, APSS and the Eaton Lab at the University of Regina commenced a community-based research partnership for a rapid assessment and response system for harm reduction and HIV and sexually transmitted and blood borne infection (STBBI) prevention [38]. These reports allow for the creation of geospatial data that can be utilized to map community needle incidence. Over time, these data can identify discarded used needle hotspots in Regina, enabling targeted harm reduction interventions, including naloxone training in areas of highest need. By integrating community-sourced data with geospatial analysis, ReportNeedles.ca enhances the ability to deploy resources and develop targeted harm reduction strategies where they are most needed. We explore the integration of ReportNeedles.ca into harm reduction strategies in Regina, focusing on three key areas: needle collection, geospatial mapping of community needle incidence, and targeted naloxone training. Through a comprehensive analysis of the data collected via ReportNeedles.ca and its application in harm reduction, this study aims to provide insights into the effectiveness of data-driven interventions in mitigating the impacts of the opioid crisis.

## Methods

### Data sources

Data sources include geospatial needle reports and questionnaires administered at pop-up naloxone trainings.

## Needle collection

Needles are reported by people in the community through the website ReportNeedles.ca. When reporting needles individuals fill out a form including a map where they are asked to drag a marker as close as possible to the needles, a location, description, and photo. Reported needles submitted through ReportNeedles.ca are reviewed by staff at APSS to ensure the report was in the catchment area. Upon reviewing a submission, APSS would deploy staff to retrieve the discarded needles regularly.

## Geospatial data

Geospatial data were created from reported needles and needle pickup data derived from ReportNeedles.ca. The data included the location of where discarded needles were found in Regina, Saskatchewan. When submitting a report, users are asked to place a marker on an interactive map to indicate the location of a discarded needle and/or provide an address. These submissions generate geographic coordinates (latitude and longitude) associated with each report. The coordinate data were imported into ArcGIS, where each report was represented as a point feature. Additional attributes (e.g., number of needles collected, date of report) were linked to each point. These data points were then used to create spatial visualizations of needle distribution and to conduct buffer analyses assessing proximity to harm reduction services.

### Pop-up naloxone trainings

Pop-up naloxone training sites were selected through a geospatial analysis of community-reported discarded needles as captured by the ReportNeedles.ca platform. Geospatial clustering of reported needles was operationalized using a descriptive and practice-oriented approach rather than statistical hotspot analysis. Reported needles were visually examined to identify areas with repeated or dense concentrations of needle reports over time. Clusters were used to describe locations with multiple reports within close geographic proximity (e.g., within the same street or small area). To support intervention planning, reports were reviewed in aggregate using both spatial proximity and frequency. Areas with higher reports of reported needles and needles recovered were prioritized for pop-up naloxone training. While no numerical threshold was used to define a cluster, decisions were guided by stability of reporting over time, and the feasibility of deploying outreach services in those locations. As a result, pop-up naloxone trainings were delivered in these areas to increase accessibility of services in areas that demonstrate the potential for higher need.

Between August 2023 and August 2024, a total of seven pop-up naloxone trainings were conducted at multiple locations across Regina, Saskatchewan. The pop-up trainings occurred at locations based on maps of community needle prevalence, including indoors at shopping centres and outdoors in parks. Trainings were held in different locations during the study period and repeatedly at the same site depending on need (based on the maps) and access to sites. The APSS team would arrive and setup the training area with signage and resources, then begin the training after approximately 15 min had passed as people congregated to participate. The trainings were facilitated by APSS staff and took approximately 5–8 min and focused on an overview of fentanyl overdose risks, recognition of overdose symptoms, overdose response strategies, and administration of naloxone. Recruitment was conducted via word of mouth and on site targeting people already present in the area. Groups varied by session, with approximately 5–10 participants attending each pop-up training.

Data were collected from 44 participants who completed pop-up naloxone trainings between August 2023 and August 2024. Survey data were collected at pop-up sites with pen and paper and later inputted and managed using Qualtrics software, a cloud-based software platform that allows for the creation, storage, and analysis of surveys. All participants who participated in Naloxone trainings completed a survey. By completing the survey, participants were provided with an honorarium of $10 Canadian. The study received ethics approval from the Research Ethics Board at the University of Regina (#236), and informed consent was obtained from all participants verbally.

## Measures

**Naloxone knowledge.** A modified version of the Opioid Overdose Knowledge Scale (OOKS) was administered to evaluate knowledge of opioid overdose risk factors and deployment of naloxone [39]. The OOKS consists of 61 true-false questions regarding (1) risk factors of overdose; (2) signs of overdose; (3) appropriate actions to take when managing an overdose; (4) knowledge of naloxone; and (5) administration of naloxone. Questions were re-worded with community consultation to focus on fentanyl usage which is more relevant to the current opioid crisis in Saskatchewan, Canada.

**Sociodemographic variables.** Sociodemographic variables included race/ethnicity, gender (cisgender woman, cisgender man, transgender and gender expansive), sexual orientation, and housing.

## Analysis

The analysis involved the creation of maps to explore geographic clusters of discarded needles in Regina, a non-walkable city [38]. All spatial analyses and map visualizations were conducted using ArcGIS. Data representing reported needle locations were imported as a feature layer using geographic coordinates (latitude and longitude). For visualization of needle distribution, a map was created using the symbology function in ArcGIS. Where multiple needles were reported at the same or near-identical coordinates, counts were aggregated and symbol size was scaled based on the number of reported needles associated with each location. Spatial patterns were interpreted descriptively based on the distribution rather than statistical hotspot testing. This approach was selected to support rapid, practice-oriented decision-making and intervention planning, rather than to estimate statistically significant spatial clusters. To examine the coverage of care and services, distance and walk time will be used as a proxy for access to services. A buffer analysis was conducted to create zones around APSS using walk time. To examine proximity to care walk time buffers were created within the ArcGIS software. Walk-time buffers were generated using the “generate travel tool” feature in ArcGIS. A 15- minute walk buffer was created for APSS and pop-up sites. This approach of creating buffers allows us to analyze the accessibility of these centers based on walk time. Walk time was selected given the non-walkable, car-centric nature of Regina and known transportation barriers faced by people who use drugs. Walk time buffers were used as a proxy for low-barrier geographic accessibility to harm reduction services, representing distances that could reasonably be traversed without reliance on private transportation. The 15-minute walk buffer was not intended to model planned attendance or intentional travel to the pop-up interventions. Rather, it functions as a geospatial proxy for the spatial reach and low-barrier availability of temporary harm reduction services within the built environment. In place-based public health research, walk-time buffers are commonly used to estimate practical accessibility and service exposure, even when services are opportunistic or embedded within everyday activity spaces. The buffer allows us to quantify the geographic extent to which services overlapped with areas of demonstrated need. The walk buffer represents a descriptive spatial measure of potential service availability relative to pedestrian presence, not an assumption about travel behaviour or program uptake. Although Regina is car-dependent, access to vehicles cannot be assumed for populations experiencing poverty, housing instability, or legal barriers to driving. This framing supports harm reduction principles that prioritize meeting people in the spaces they already occupy. The buffer is not intended to model actual travel behaviour, but rather to estimate the spatial reach of services relative to everyday pedestrian presence. Walk buffers were selected as an interpretable metric suited to real-time intervention planning rather than formal transportation modeling. Additionally, a 15-minute walk buffer was selected to represent a pragmatic proxy for low-barrier access to harm reduction services. This threshold is particularly relevant for a population with limited resources and health and mobility constraints. Descriptive statistics using proportions were used to summarize Naloxone trainee knowledge. Geospatial analysis was conducted using ArcGIS and statistical analysis was conducted using R Statistical Software. Descriptive statistics using means, standard deviations, and frequencies were used to describe the sample.

## Results

**Fig. 1:**
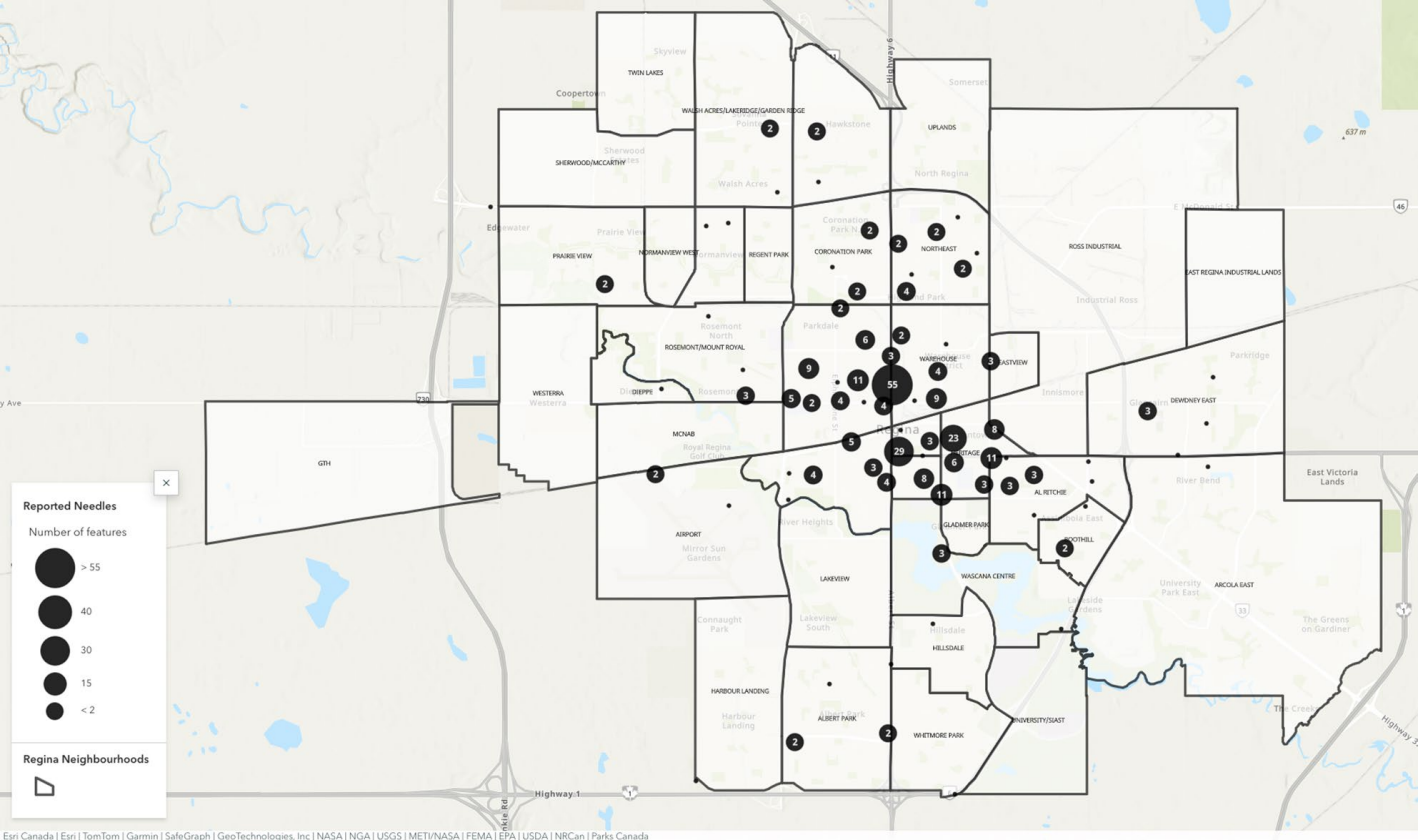
Spatial distribution of reported needles in Regina, Saskatchewan, Canada, between August 2023 and August 2024. Each point represents a reported needle location recorded through the ReportNeedles.ca platform. Circle size corresponds to the number of reported needles per location, with larger symbols indicating higher counts

**Fig. 2:**
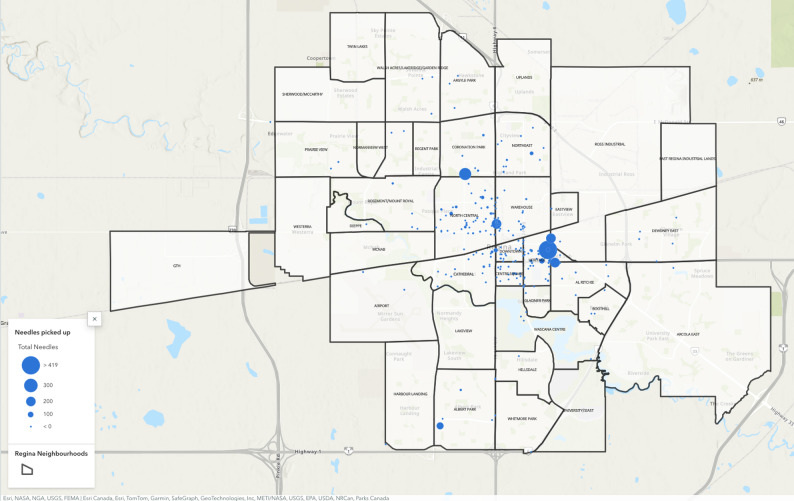
Spatial distribution of reported needle pickup counts in Regina, Saskatchewan, Canada, between August 2023 and August 2024. Each point represents a location where reported needles were retrieved following community submissions to the ReportNeedles.ca platform. Circle size corresponds to the number of needles collected per location, with larger symbols indicating higher retrieval counts

Between August 2023 and August 2024, a total of 328 reports were submitted on reportneedles.ca, of which 315 were successful (~ 96% success rate) resulting in the disposal of 2,836 needles (see Figs. 1 and 2). The mapping of reported needles revealed spatial clustering of discarded needles (see Fig. 1). This map shows the clustering of reported needles in particular neighbourhoods focused in the city centre such as Warehouse (*n* = 62), North Central (*n* = 57), Heritage (*n* = 28), Downtown (*n* = 12). However, needles were also reported in the surrounding suburbs. Similarly, the number of needles disposed of were highest in the city centre (See Fig. 2).

**Fig. 3:**
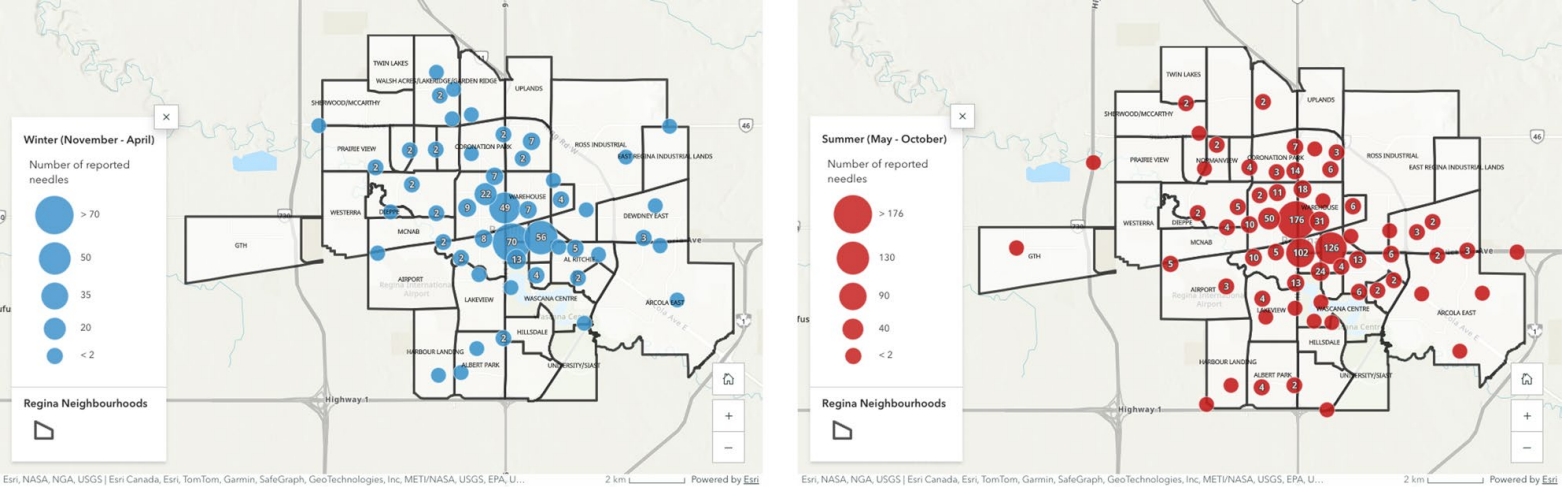
Spatial distribution of reported needles in Regina, Saskatchewan, Canada, by season during August 2023–August 2024. The left panel displays reported needles during winter months (November–April), and the right panel displays reported needles during summer months (May–October). Each point represents a reported needle location recorded through the ReportNeedles.ca platform. Circle size corresponds to the number of reported needles per location, with larger symbols indicating higher counts

Between two 6-month periods (Winter months [November - April] vs. summer months [May - August]) we examined how needles report locations changed (See Fig. 3). Based on visual inspections of the maps it appears that discarded needles were still primarily reported in the city centre. However, during the summer months it appears that there is more variation in where needles are reported and multiple reports in suburban areas. Whereas, in the winter the needle reports were more concentrated in the city centre with few reports in suburban areas.

**Fig. 4:**
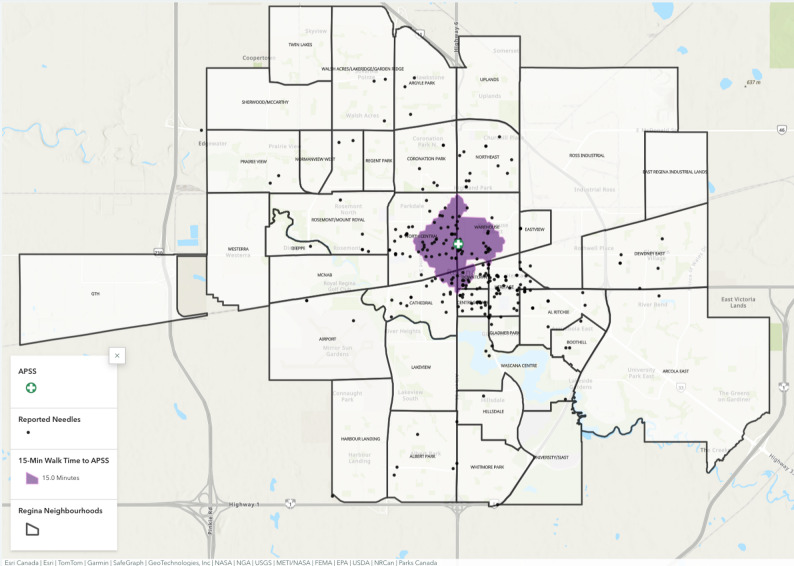
Spatial distribution of reported needle locations and 15-minute walk buffer to APSS in Regina, Saskatchewan, Canada, during August 2023–August 2024. Black points represent individual reported needle locations recorded through the ReportNeedles.ca platform. The shaded area indicates the 15-minute walk-time buffer surrounding APSS, representing the geographic area accessible by walking within 15 min

**Fig. 5:**
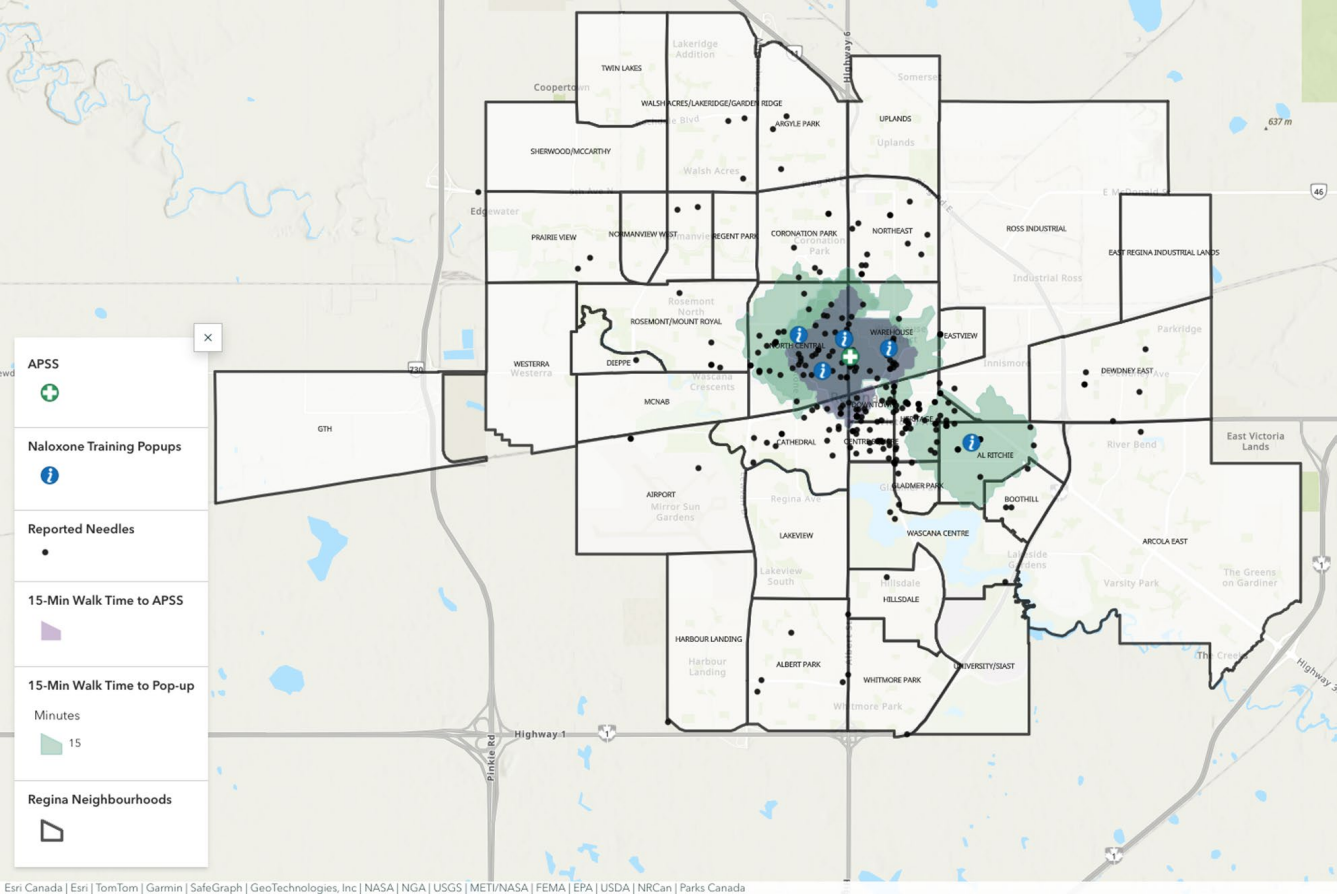
Spatial distribution of reported needle locations and 15-minute walk buffer to APSS and pop-up sites in Regina, Saskatchewan, Canada, during August 2023–August 2024. Black points represent individual reported needle locations recorded through the ReportNeedles.ca platform. The shaded purple area indicates the 15-minute walk-time buffer surrounding APSS and the shades green areas indicates the 15-minute walking buffer surrounding the pop-up sites, representing the geographic area accessible by walking within 15 min

Access to care was analyzed by examining a 15-minute walk time buffer to APSS and pop-up trainings (see Figs. 4 and 5). Based on visual inspections of the maps examining proximity to care the pop-up trainings allowed APSS to expand the reach of their services. Of the 315 reported needle locations, 122 (38.7%) were located within a 15-minute walk APSS, whereas 245 (77.8%) were located within a 15-minute walk of either APSS or a pop-up naloxone training site, indicating that pop-up interventions substantially expanded geographic service coverage. Currently APSS is accessible by a 15-minute walk to primarily three neighbourhoods and is not accessible to many areas where needle have been reported. By expanding and offering pop-up sites for naloxone trainings APSS is able to expand the accessibility of their services to cover more neighbourhoods and area. However, given the large number of needles and the city centre and limited resources naloxone trainings were not held in suburban areas.

A total of 7 pop-up trainings occurred between August 2023 and September 2024. Pop-up trainings were focused on areas with high needle prevalence. A total of 44 participants were trained in overdose recognition and naloxone administration between August 2023 and September 2024 (See Table 1 for full participant demographics). Participants were predominantly cisgender women (61.4%; *n* = 27) or cisgender men (29.5%; *n* = 13). The majority of participants were Indigenous (63.7%; *n* = 28) or White (36.4%; *n* = 16). The participants who attended the training had a mean age of 41.6 (SD = 14.6; Range: 17–73). Approximately 70% of the sample reported that the harm reduction centre was within a 15-minute walk of their residence.

**Table 1:** Participant Demographic Characteristics (*N* = 44)

Characteristic	*n* (%) or Mean (SD)
**Age**	41.6 (SD = 14.6)
Gender ^1^	
Cisgender women	27 (61.4%)
Cisgender men	13 (29.5%)
Transgender and gender diverse	3 (6.8%)
**Race/Ethnicity** ^**2**^	
Indigenous (First Nations, Inuit, Métis)	28 (63.7%)
White	16 (36.4%)
Other racialized group	6 (13.6%)
**Is the pop-up location within a 15 min walk?**	
Yes	31 (70.5%)
No	13 (29.5%)

¹ Gender identity categories with small cell sizes were collapsed for reporting to improve interpretability and reduce the risk of participant identifiability. The category Transgender and gender-diverse includes transgender women, transgender men, and Two-Spirit participants.

² Race/ethnicity categories were not mutually exclusive; therefore, percentages may exceed 100%. Race/ethnicity categories with small cell sizes were collapsed for reporting to improve interpretability and reduce the risk of participant identifiability. The category Other racialized groups includes Black, Latino/a, East/Southeast Asian, and South Asian participants.

Results from the OOKS showed that participants were knowledgeable on opioid overdose and naloxone administration (See Table 2 for training results). The majority of participants (70.2%) correctly determined the risk factors of an opioid overdose. Training participants were largely successful at indicating signs of an opioid overdose (75.6%) but struggled to discern at least one incorrect symptom *(*39.3%). Regarding naloxone administration, the majority of participants were able to recognize the appropriate actions to take (83.7%) and inappropriate actions to avoid (93.8%) when managing an opioid overdose. While **a** majority of the sample indicated the correct use of naloxone for opioid overdose (90.9%), a greater proportion erroneously suggested that naloxone could also reverse the effects of a stimulant (95.5%) and Benzodiazepines (93.2%). Over 93% of participants successfully identified the administration route of naloxone and over 90% understood that naloxone injection should be delivered through thighs or upper arms. The majority of the sample accurately determined the length of onset (81.8%) and duration of naloxone (61.4%).

**Table 2:** Opioid Overdose Knowledge Scale Items (*n* = 44)

Risk Factors of Opioid Overdose Risk	*N* Correct	% Correct
Which of the following factors increase the risk of a fentanyl (opioid) overdose?		
Taking larger than usual doses of fentanyl. (T)	36	81.8%
Switching from smoking to injecting fentanyl (T)	28	63.6%
Using heroin with other substances, such as alcohol or sleeping pills (T)	32	72.7%
Increase in fentanyl purity (T)	27	61.4%
Using fentanyl again after not having used for a while (T)	35	79.5%
Using fentanyl when no one else is present around (T)	31	70.5%
A long history of fentanyl use (T)	30	68.2%
Using heroin again soon after release from prison or from a detox treatment (T)	28	63.6%
*Signs of overdose*		
Having blood-shot eyes (F)	9	20.5%
Slow/shallow breathing (T)	39	88.6%
Lips, hands or feet turning blue (T)	39	88.6%
Loss of consciousness (T)	39	88.6%
Unresponsive (T)	39	88.6%
Seizures or convulsions (T)	16	36.3%
Deep snoring (T)	27	61.4%
Very small pupils (T)	34	77.3%
Agitated behaviour (F)	16	36.4%
Rapid heartbeat (F)	18	60.9%
**Naloxone knowledge and administration**		
*Which of the following should be done when managing an opioid overdose?*		
Call an ambulance (T)	43	97.7%
Stay with the person until an ambulance arrives. (T)	39	88.6%
Inject the person with salt solution or milk. (F)	42	95.5%
Mouth to mouth resuscitation. (T)	28	63.6%
Give stimulants (e.g. cocaine or black coffee). (F)	40	90.9%
Place the person in the recovery position (on their side with mouth clear).	35	79.5%
Give Naloxone (opioid antidote). (T)	41	93.2%
Put the person in a bath of cold water. (F)	40	90.9%
Check for breathing. (T)	37	84.1%
Check for blocked airways (nose and mouth). (T)	35	79.5%
Put the person in bed to sleep it off. (F)	43	97.3%
*What is naloxone used for?*		
To reverse the effects of a fentanyl overdose (e.g. heroin, methadone). (T)	40	90.9%
To reverse the effects of a stimulant (e.g., crystal meth, cocaine) overdose. (F)	42	95.5%
To reverse the effects of Benzodiazepines (e.g. Xanax, Ativan. (F)	41	93.2%
*How can naloxone be administered?*		
Into a muscle (intramuscular) (T)	41	93.2%
Into a vein (intravenous) (T)	2	4.5%
Under the skin (subcutaneous) (T)	4	9.1%
Swallowing liquid/tablet (F)	43	97.3%
*Where is the most recommended place for non-expert to administer naloxone?*		
Outside of thighs or upper arms (T)	40	90.9%
Any vein (F)	43	97.3%
Heart (F)	0	0%
By mouth (F)	43	97.3%
*How long does naloxone take to start having an effect?*		
2–5 min (T)	36	81.8%
6–10 min (F)	4	9.1%
11–20 min (F)	1	2.3%
21–40 min (F)	0	0%
*How long do the effects of naloxone last for?*		
Less than 20 min (F)	5	11.4%
About 1 h (T)	27	61.4%
1 to 6 h (F)	3	6.8%
6 to 12 h (F)	0	
*Tick each correct statement*		
If the first dose of naloxone has no effect a second dose can be given (T)	38	86.4%
There is no need to call for an ambulance if I know how to manage an overdose (F)	38	86.4%
Someone can overdose again even after having received naloxone (T)	37	84.1%
The effect of naloxone is shorter than the effect of heroin and methadone (T)	21	47.7%
After recovering from an opioid overdose, the person must not take any heroin, but it is OK for them to drink alcohol or take sleeping tablets (F)	42	95.5%
Naloxone can provoke withdrawal symptoms (T)	21	47.7%

T = True; F= False

## Discussion

The study results suggest that mapping of community needles and pop-up brief opioid education and naloxone training interventions are a feasible approach for delivering overdose prevention education and naloxone training in community settings. Given the absence of pre-intervention or follow-up assessments, these findings should be interpreted as descriptive of participant knowledge at the time of training rather than as evidence of changes attributable to the intervention. Following the training participants demonstrated strong knowledge of opioid overdose and awareness of appropriate procedures to follow in the event of an overdose. Recent studies corroborate findings that naloxone trainings significantly improve participants’ awareness, knowledge, and confidence regarding overdose management [27, 28, 40]. Prior research suggest that participants show consistent retention of overdose management knowledge of up to 6 months after interventions [22]. Additionally, prior research has shown that naloxone trainings are effective in a diverse range of populations including those who are racialized and experiencing homelessness [26, 27]. Furthermore, the primary goal of this study is not to evaluate training outcomes, but to demonstrate the feasibility of using real-time, community-generated geospatial data to guide the placement and delivery of pop-up harm reduction interventions.

In our study, multiple naloxone training sessions occurred on or within walking distance of an urban reserve in Regina. This enabled the pilot study to successfully recruit a majority Indigenous sample, fostering skills in overdose management and naloxone administration in a community that is disproportionately vulnerable to drug-related harms. Prior research has shown that Indigenous Canadians are more likely to have opioids use disorders, and are at higher risk of drug-toxicity deaths, and are more vulnerable to HIV/STBBI infections, but reportedly view harm reduction programming as culturally incompatible [13, 14]. In the literature, few studies appear to specifically focus on naloxone training initiatives directed at Indigenous populations. Levine et al. describes how Indigenous-led “Not-Just-Naloxone” workshops in British Columbia provided culturally relevant harm reduction education and training, increasing knowledge, awareness, and self-efficacy among First Nations participants [40].

In our study, most participants lived within walking distance of the pop-up naloxone trainings. Prior research has found that transportation is a common barrier to the initiation and engagement of substance use treatment [41, 42]. Regina is a non-walkable, car-centric city, which limits the ability of made marginalized populations to access healthcare services [38]. As depicted in the map data, there are moderate levels of community needle prevalence in Regina’s surrounding neighbourhoods, far from the city’s harm reduction services. Conversely, approximately 70% of this study’s sample reported that the pop-up naloxone training session was within a 15-minute walk to their residence. Pop-up interventions may increase access to and uptake of harm reduction services among underserved communities with limited transportation options.

GIS mapping in the health science literature has been utilized to map the distribution of resources and identify regional health inequities [33, 35, 37, 43]. While prior studies have been largely retrospective and have not integrated GIS into health programming, the findings of this study demonstrate that real-time geospatial data on community needle prevalence can successfully facilitate targeted harm reduction interventions in areas of highest need. Additionally, previous studies, particularly in Canada, have largely mapped overdose-related data onto cities [44, 45]. Geospatial analysis enables the creation of tailored community-based interventions for underserved populations, specifically people who use drugs, who may not access conventional healthcare services for a variety of reasons. People who use drugs who are transient have barriers to accessing healthcare [35, 37]. Geospatial analysis of needle prevalence data enables health interventions to adapt to the migratory patterns of transient populations. GIS-informed, community-based education, screening, and treatment interventions may increase engagement in care as well as reduce rates of overdose and HIV/STBBI transmission.

Future directions on this research should focus on naloxone misconceptions to improve training outcomes. A large proportion of participants in this study incorrectly reported that naloxone can be used to reverse a stimulant or Benzodiazepine-related overdose. Additionally, many participants erroneously indicated that calling an ambulance is unnecessary after administering naloxone, which may be attributed to poor experiences with healthcare and policing among PWUD and Indigenous peoples [43, 44]. Belief in naloxone misconceptions may result in inappropriate and detrimental use. Furthermore, neglecting to alert emergency services after an overdose may lead to the oversight of overdose-related complications, such as heart irregularities and pneumonia [45]. Future research can investigate the prevalence of naloxone misconceptions among people who use drugs and guide OEND interventions to dispel these myths. Further work is also needed to rebuild the relationship between healthcare services, police, and PWUD, specifically Indigenous individuals who use substances. More naloxone trainings should be targeted for Indigenous populations in Canada, who are at higher risk of overdose. Indigenous populations in Canada are overrepresented within drug toxicity deaths, HIV/STBBI infections, and Hepatitis C cases.

[8, 46]. Additionally, greater involvement of Indigenous people, specifically those with lived and living experience of drug use, within research is essential in developing culturally tailored initiatives and increasing engagement in preventative health services. Utilizing GIS analysis to target interventions to underserved populations, specifically people who use drugs is a novel approach that builds on previous work using geospatial methods to examine health disparities and spatial inequities in service access.

Prior research has demonstrate the utility of GIS for identifying geographic concentrations of unmet health needs and informed targeted public health responses [31, 32, 34, 35]. More recently, studies have applied geospatial approaches to guide HIV/STTBI testing interventions [47]. This present study extends this literature by demonstrating how real-time, community generated needles reporting data can be operationalized to guide harm reduction interventions to reach transient, marginalized populations. Future studies may explore the application of geospatial analysis for other infectious diseases and health interventions. Additionally, there is a limited focus on the suburbs (i.e., residential areas that are located on the outskirts or periphery of a central city or urban area.) in the literature. Drug-related harms may be overlooked in these regions, even though these communities have similarly limited access to harm reduction supports as more rural areas. Pop-up interventions can expand the reach of harm reduction organizations and improve the availability of overdose prevention services for “hidden” populations in need.

Overall, these findings have important implications for harm reduction research, practice, and policy. From a research perspective, this study demonstrates the feasibility of integrating community generated geospatial data into real-time intervention planning. For practice, the use of pop-up naloxone trainings informed by community discarded needle data offers a flexible model for expanding access to harm reduction services in non-walkable cities particularly for people who may not be able to access brick and mortar programs. At the policy level, these results highlight the importance of investment in harm reduction program and infrastructure that account for both urban and suburban patterns of drug-related harm.

### Limitations

This study contains several limitations. Firstly, no pre-test and follow-up assessments were conducted, meaning that changes in or retention of knowledge regarding overdose and naloxone administration cannot be determined. Without a pre-test it is unclear whether participants had prior familiarity with opioid overdose management and naloxone administration making it difficult to assess the effectiveness and utility of the training. However, the focus of this study was on the implementation of the geospatial approach as prior research has shown the effectiveness of naloxone trainings. Another limitation of this study is related to sampling. The training sessions likely attracted a self-selected group of individuals who were willing and able to participate. Likewise, participation in the pop-up naloxone trainings was opportunistic and subject to selection bias, as attendees were limited to those present at pop-up sites. This bias potentially results in a group different that the broader population of people who use substances. For example, those with less familiarity with naloxone may be underrepresented. Additionally, despite the wide distribution of discarded needles in Regina suburbs, pop-up interventions were limited to areas of highest need, due to limited resources. Correspondingly, reported needle locations reflect not only patterns of drug use, but also visibility, pedestrian activity, and reporting behavior. For instance, seasonal variation in reporting, particularly fewer winter reports in suburban areas, may reflect reduced pedestrian activity and snow cover rather than true declines in needle disposal. Furthermore, the research team did not inquire participants on their history of drug use. Therefore, while the pop-up naloxone training sessions aimed to reach individuals who use substances in areas of high drug activity, it remains unclear whether people who use drugs, who are best positioned to benefit from naloxone training and reverse overdoses, engaged in the OEND interventions. Furthermore, this study was not designed to produce population-representative estimates of needle prevalence or naloxone knowledge among all residents of Regina but rather to examine feasibility and implementation among people present in public spaces within neighbourhoods experiencing elevated drug-related harms. Lastly, ReportNeedles.ca is a tool that depends on user reports of discarded needles in the community. Consequently, ReportNeedles.ca may be impacted by reporting bias and underreporting, since users may be unequally distributed within Regina, leading to certain regions having more comprehensive and up-to-date data than other areas [33]. For example, in a car-dependent city such as Regina, suburban areas with lower walkability and greater reliance on vehicle travel may be underrepresented.

## Conclusion

This article presents promising results from a geospatial, community-based needle collection and naloxone training initiative. In one non-walkable, medium-sized Canadian city, injection drug use is dispersed throughout the city and outside walking distance to healthcare and harm reduction services. Developing strategies for culturally tailored, geo-located street-outreach may help intervene during drug-related crises.

## Data Availability

No datasets were generated or analysed during the current study.

## Abbreviations

OEND: Opioid Overdose Prevention Education and Naloxone Distribution
HIV: Human Immunodeficiency Virus
STBBI: Sexually Transmitted and Blood-Borne Infections
